# ^18^F-FDG and ^18^F-FLT-PET Imaging for Monitoring Everolimus Effect on Tumor-Growth in Neuroendocrine Tumors: Studies in Human Tumor Xenografts in Mice

**DOI:** 10.1371/journal.pone.0091387

**Published:** 2014-03-13

**Authors:** Camilla Bardram Johnbeck, Mette Munk Jensen, Carsten Haagen Nielsen, Anne Mette Fisker Hag, Ulrich Knigge, Andreas Kjaer

**Affiliations:** 1 Department of Clinical Physiology, Nuclear Medicine & PET and Cluster for Molecular Imaging, Faculty of Health Sciences, Rigshospitalet and University of Copenhagen, Copenhagen, Denmark; 2 Department of Surgical Gastroenterology C and Department of Endocrinology, Rigshospitalet, Copenhagen, Denmark; NIH, United States of America

## Abstract

**Introduction:**

The mTOR inhibitor everolimus has shown promising results in some but not all neuroendocrine tumors. Therefore, early assessment of treatment response would be beneficial. In this study, we investigated the *in vivo* and *in vitro* treatment effect of everolimus in neuroendocrine tumors and evaluated the performance of ^18^F-FDG and the proliferation tracer ^18^F-FLT for treatment response assessment by PET imaging.

**Methods:**

The effect of everolimus on the human carcinoid cell line H727 was examined *in vitro* with the MTT assay and *in vivo* on H727 xenograft tumors. The mice were scanned at baseline with ^18^F-FDG or ^18^F-FLT and then treated with either placebo or everolimus (5 mg/kg daily) for 10 days. PET/CT scans were repeated at day 1,3 and 10.

**Results:**

Everolimus showed significant inhibition of H727 cell proliferation *in vitro* at concentrations above 1 nM. *In vivo* tumor volumes measured relative to baseline were significantly lower in the everolimus group compared to the control group at day 3 (126±6% vs. 152±6%; p = 0.016), day 7 (164±7% vs. 226±13%; p<0.001) and at day 10 (194±10% vs. 281±18%; p<0.001). Uptake of ^18^F-FDG and ^18^F-FLT showed little differences between control and treatment groups, but individual mean uptake of ^18^F-FDG at day 3 correlated with tumor growth day 10 (r^2^ = 0.45; P = 0.034), ^18^F-FLT mean uptake at day 1 correlated with tumor growth day 7 (r^2^ = 0.63; P = 0.019) and at day 3 ^18^F-FLT correlated with tumor growth day 7 (r^2^ = 0.87; P<0.001) and day 10 (r^2^ = 0.58; P = 0.027).

**Conclusion:**

Everolimus was effective *in vitro* and *in vivo* in human xenografts lung carcinoid NETs and especially early ^18^F-FLT uptake predicted subsequent tumor growth. We suggest that ^18^F-FLT PET can be used for tailoring therapy for neuroendocrine tumor patients through early identification of responders and non-responders.

## Introduction

Neuroendocrine tumors (NETs) are a heterogeneous group of tumors derived from neuroendocrine cells mainly from the gastrointestinal tract and the bronchopulmonary system. NETs have been considered a rare type of cancer but according to epidemiological data, the incidence has increased five-fold from 1.09 to 5.25/100.000 from 1973 to 2004. Since the survival has increased as well, gastrointestinal NET is now the second most prevalent cancer in the gastrointestinal tract [1].

The treatment of NETs has for many years been multifaceted including surgery, somatostatin analogs, interferon alfa, chemotherapy, and peptide receptor radionuclide therapy [2], [3]. Currently, new targeted therapies and combinations of known treatments are being tested. Accordingly, targeted therapies with everolimus and sunitinib have recently been approved for subgroups of NETs [4].

The gold standard to monitor chemotherapy response in solid tumors has for more than a decade been measurements of the tumor size by CT using the RECIST criteria [5], [6]. A problem with this method arises when the treatment, as intended, causes cell death and necrosis but does not cause immediate shrinkage in tumor size. This is a well-described phenomenon in several forms of neoplasms and especially when new targeted treatments that are not cytotoxic are being used [7], [8].

At the same time, evidence gained from several tumor types show that additional information is obtained when Positron Emission Tomography (PET) is used for treatment monitoring compared to CT measurements alone [9]–[11]. Monitoring the functional status of tumors by PET imaging during treatment has shown the ability to predict a positive treatment-response at an earlier stage compared to volume based CT-response [12]–[16].

Most widely used in cancer imaging and treatment monitoring is PET using ^18^F-fluorodeoxyglucose (FDG).

FDG uptake reflects cancer cell glycolysis and has been shown to correlate with early treatment responses both in preclinical studies and patient studies although not in all cases [17].

Another hallmark of cancer, proliferation, can be measured using ^18^F-fluorothymidine (FLT) [18]. FLT is a thymidine analogue and mimics the nucleosides used for DNA synthesis [19]. FLT is taken up by the cell by nucleoside transporters and is phosphorylated by thymidine kinase 1 (TK1), which leads to intracellular trapping. Uptake of FLT is assumed to reflect the amount of proliferating cells since TK1 is mainly expressed during the S-phase of the cell cycle [20], [21]. Several studies have found a correlation between uptake of FLT and tumor cell proliferation measured by Ki67 [22]. In a meta analysis of 27 studies, the FLT/Ki67 correlation was found to be strong and independent of cancer-type [23]. The use of FLT for early treatment monitoring has like FDG been promising for some tumors and less usable for others [16], [24]–[26]. The conclusion when reviewing FDG and FLT for the purpose of response monitoring is the importance of using a reproducible method. It seems reasonable though that the tracers might be useful for some tumor and treatment types and not for others [10].

Everolimus is an inhibitor of the mammalian target of rapamycin (mTOR), a downstream component of the PI3K/AKT pathway involved in the regulation of cell proliferation, metabolism and angiogenesis.

It is known that the mTOR pathway is dysregulated in some NETs due to aberrant events such as loss or down-regulation of the tumor suppressor gene PTEN or tuberous sclerosis 2 (TSC2). Low TSC2 and PTEN are linked to progression of the cancer [27] and although the molecular pathogenesis of sporadic pancreatic NETs is unknown, several genetic cancer syndromes involving the mTOR pathway, including tuberous sclerosis, neurofibromatosis, and von Hippel–Lindau disease, are linked to the development of pancreatic NETs [28].

In line with this, everolimus has recently been approved by FDA and EMA for the treatment of advanced pancreatic NETs.

At the same time, it is becoming evident that subgroups of NET-patients for some reason have a worse prognosis than others. FDG has shown to be highly prognostic for NET-patients across the classification system [29]. The need for individual monitoring of the response to the diversity of treatments is thus increasingly important.

Since everolimus has shown promising results in some NETs and the antitumor effect is mediated through inhibiting the mTOR complex, it seems likely that tracers detecting glycolysis and proliferation could serve as early predictors of a treatment response or failure.

In the present study, we monitored the response to everolimus treatment of NET *in vitro* and in an animal model using FDG and FLT PET. The aim was to evaluate the effect of everolimus in NET and if FDG/FLT PET imaging could be used as an imaging biomarker for early prediction of the treatment response.

## Materials and Methods

### Ethics Statement

Animal care and experimental procedures were approved by the Danish Animal Welfare Council (license no. 2011/561-14).

### Cell Line used for *in vitro* and *in vivo* Study

The human neuroendocrine tumor cell-line, lung bronchus carcinoid, NCI-H727 obtained from ATCC (American Type Culture Collection, Manassas, VA, USA) was used. The cell line was tested to be free of Mycoplasma at Statens Serum Institute, Copenhagen, Denmark. The cells were cultured in RPMI Medium 1640 with GlutaMAX™ (Gibco, Life Technologies, NY, USA) containing 10% fetal calf serum (Biological Industries, Kibbutz Beit-Haemek, Israel) and 1% penicillin-streptomycin (Gibco, Life Technologies) in 5% CO_2_ at 37^0^C.

### MTT Assay

H727 cells were plated in flat bottom 96-well plates at 3000 cells/well and incubated overnight. Treatment with everolimus was done in triplicates at escalating concentrations from 1 to 1000 nM for 72 hours. The cell proliferation was monitored with the MTT assay according to manufacturer protocol (Vybrant MTT Cell Proliferation Assay Kit, Invitrogen, Naerum, Denmark) and the proliferation was normalized to the untreated cells.

### *In vivo* Study

**Figure 1:**
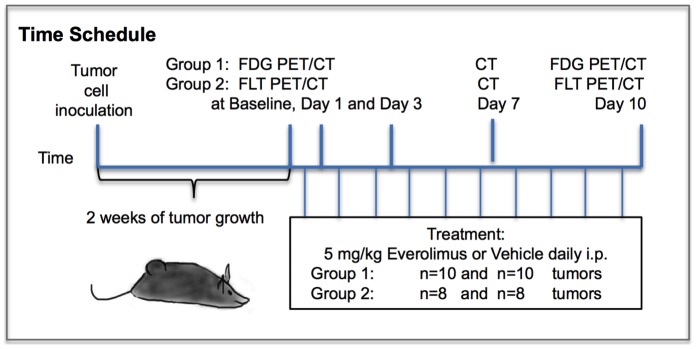
Study design.

The study design is illustrated in Figure 1. Twenty 6 weeks old female NMRI (Naval Medical Research Institute) nude mice were acquired from Taconic Europe, (Lille Skensved, Denmark). After 1 week of acclimatization, the mice were inoculated with H727 cells (5–7×10^6^ cells in 100 uL medium mixed with 100 uL Matrixgel™ Basement Membrane Matrix; BD Sciences, San José, CA, USA). The inoculates were injected subcutaneously on the left and right flank during anesthesia with 1∶1 V/V Hypnorm^R^ (Janssen Pharmaceutica NV, Beerse, Belgium)/Dormicum^R^ (Roche, Basel, Switzerland) i.p. Tumors were allowed to grow for 2 weeks. Half of the mice were FLT-PET scanned and the other half were FDG-PET scanned. At day 0 “baseline” examinations with FDG or FLT PET-scans as well as CT-scans were performed. Thereafter we randomized the mice to treatment with everolimus (n = 10) or vehicle (n = 10). The mice were treated daily i.p with 0.250 ml solution, either vehicle consisting of 18 ml 5% glucose and 2 ml DMSO, or 10 mg of everolimus (Sigma-Aldrich, Copenhagen, Denmark) diluted in 18 ml 5% glucose and 2 ml DMSO. Daily dose of everolimus was 5 mg/kg [30]. The mice were sacrificed on day 10. CT scans for measurements of tumor size were performed at baseline and at day 1, 3, 7 and 10. FDG and FLT PET imaging were performed at baseline, and at day 1, 3 and 10. Twenty tumors were inoculated for each PET-scan group. At the time of PET imaging, sixteen tumors in the FLT group and 20 tumors in the FDG group were suitable for measurements.

### Synthesis of PET-tracers

^18^F-FDG was acquired from daily productions for clinical use at our facility (Rigshospitalet, Copenhagen, Denmark).

^18^F-FLT was synthesized using 3-N-Boc-1-[5-O-(4,49-dimethoxytrityl)-3-O-nosyl-2-deoxy-b-D-lyxofuranosyl]thymine as precursor and synthesized on a GE TracerLab MX Synthesizer as previously described [12].

### Imaging with microPET and microCT

Mice were injected i.v. with approximately 10 MBq of FDG or FLT. Before each FDG scan, the mice were fasted overnight. The mice were anaesthetized with 3% sevoflurane (Abbot Scandinavia AB, Solna, Sweden) mixed with 35% 0_2_ in N_2_ and fixed on a bed compatible with both the PET and CT scanner. A 15 min PET scan was acquired using a MicroPET Focus 120 (Siemens Medical Solutions, Malvern, PA, USA) [31].

The PET data were arranged into sinograms and subsequently reconstructed with the maximum a posteriori (MAP) reconstruction algorithm after data acquisition. The pixel size was 0.300×0.300×0.796 mm and average resolution was 1.4 mm full-width-at-half-maximum. The images were not corrected for attenuation or scatter [32].

Immediately following the microPET scan, a microCT scan was acquired using a MicroCAT II system (Siemens Medical Solutions) [31]. A 7 minute and 10 seconds CT scan was performed with the following parameter settings: 360 rotation steps, tube voltage 60 kV, tube current 500 mA, binning 4 and exposure time 310 ms. The pixel size was 0.0916×0.0916×0.091 mm. The mice were not moved between the scans.

Fusion of microPET and microCT images was done in the Inveon software (Siemens Medical Solutions). Before fusion, region of interests (ROIs) were drawn on the CT pictures manually by qualitative assessment covering the whole tumors and subsequently tumor volume and tracer uptake, assessed by standardized uptake values (SUV) mean and maximum, were generated by summation of voxels within the tomographic planes. SUV was defined as (CT*W)/D_inj_, where CT is radioactivity in the tissue, W is weight of the animal and D_inj_ is injected dose. SUV_mean_ gives information of the mean concentration of tracer and SUV_max_ is a measure of the voxel within the ROI that have the highest tracer concentration. For tumor volume measurements, ROIs were drawn manually by delineation of the tumor boundary by qualitative assessment in several of the coronal planes on the microCT images. Summation of voxels from all the planes generated the tumor volume. Tumor volume can be correctly assessed by this method [33].

### Statistical Analysis

Unpaired students t-test was used for comparison between treatment and control groups. Paired t-test was used for intra-group comparisons. Shapiro Wilk test for normality and Levenes test for equality of variances were used. Bonferroni correction of p-values for multiple comparisons was applied. Linear regression was used for calculation of correlations between early SUV_mean_ and tumor growth. Calculations were made in SPSS 20 (IBM Corporation, Armonk, NY, USA). Data are reported as mean ± SEM and p<0.05 was considered statistically significant.

## Results

### Everolimus and H727 Cell Proliferation

**Figure 2:**
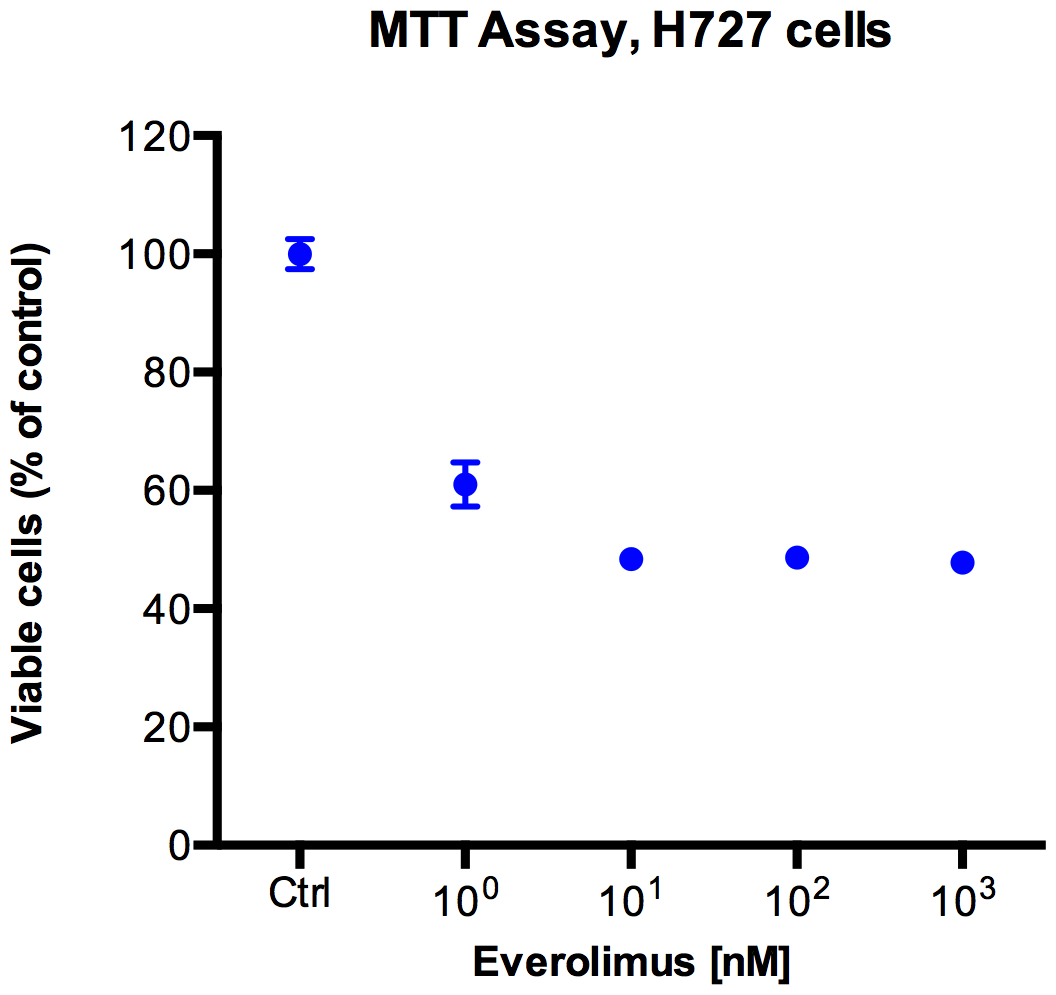
MTT Assay. *In vitro* inhibition of neuroendocrine tumor cell proliferation by everolimus using MTT assay.

Everolimus showed significant inhibition of H727 cell proliferation *in vitro* at concentrations above 1 nM (Figure 2).

### Everolimus Treatment and H727 Xenograft Tumor Growth

Treatment of H727 xenografts with everolimus 5 mg/kg daily for 10 days resulted in decreases in tumor sizes compared to the vehicle treated control group. There were 36 tumors evaluable for growth monitoring (n = 18 in each group). No significant difference between the mean baseline tumor sizes of the groups was found. The tumor size at baseline was 185±14 (range 64–354 mm^3^). The relative size of the tumors, given as the percent of baseline, was used for evaluating the tumor growth.

**Figure 3:**
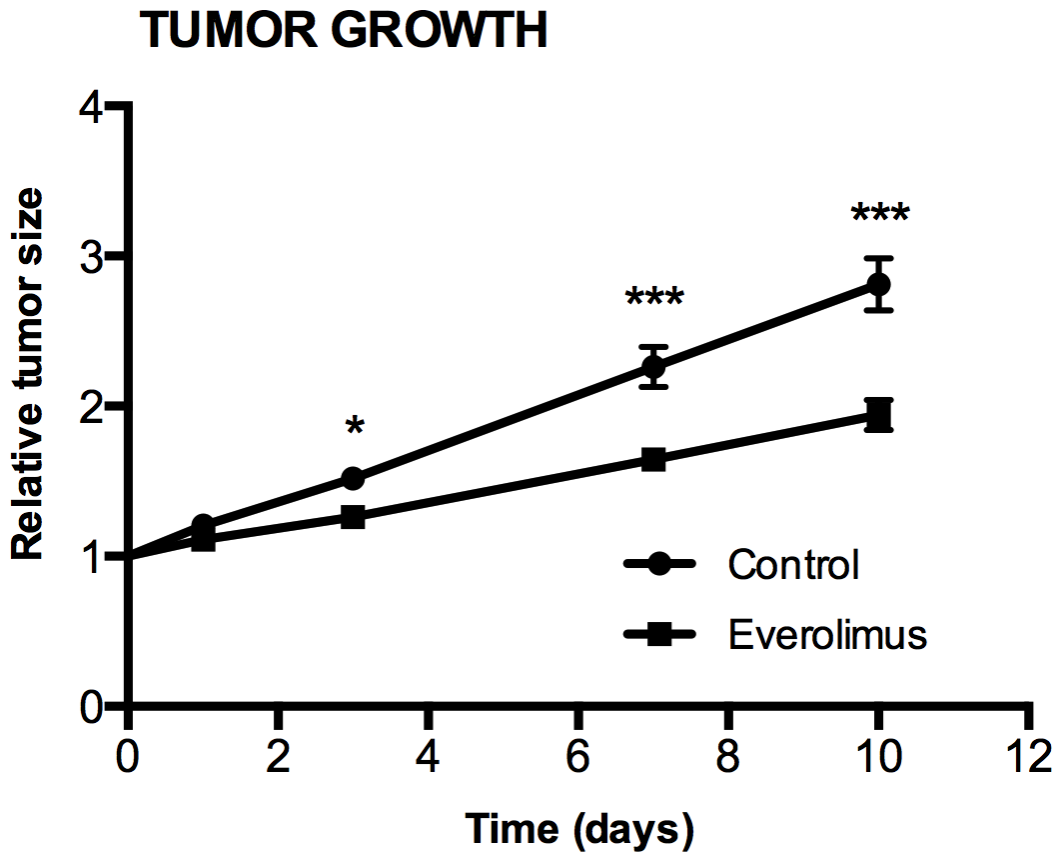
Tumor growth of everolimus and vehicle treated human H727 xenografts in nude mice. A significant effect of everolimus treatment was found on day 3, 7 and 10 compared with the control group. Tumor volume was found by manual drawing on the tumor boundary on CT images. N = 18 tumors/group. *) P<0.05, **) P<0.01, ***) P<0.001, treatment versus control group at same day.

As shown in Figure 3, treatment with everolimus resulted in significantly less tumor growth compared to the control group. Relative tumor sizes were significantly reduced in the everolimus group compared to the control group on day 3 (126±6% vs. 152±6%; p = 0.016), day 7 (164±7% vs. 226±13%; p<0.001) and day 10 (194±10% vs. 281±18%; p<0.001). At day 1 the difference was not significant (111±2% vs. 120±4%; p = 0.173).

### Uptake of FDG in H727 Xenografts and Treatment with Everolimus

**Figure 4:**
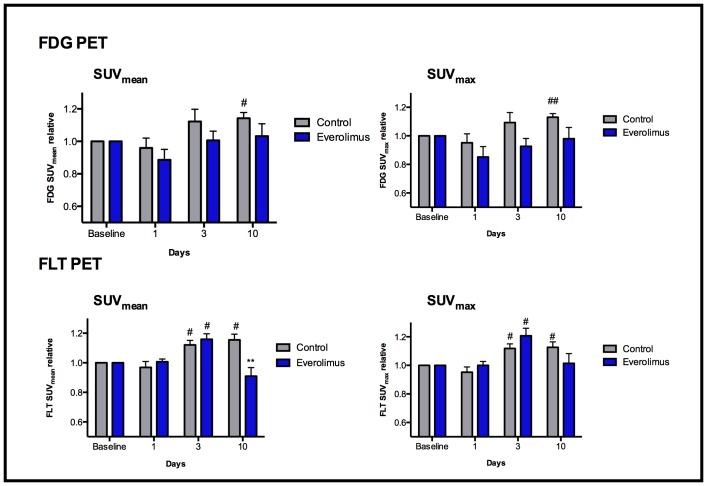
PET uptake in H727 xenografts. Uptake of FDG (upper panel) and FLT (lower panel) after treatment of H727 xenografts with everolimus or vehicle. N = 8–10 tumors/group. *) P<0.05 vs. control group on same day, #) P<0.05 and ##) P<0.01 vs. baseline of same group. P-values are Bonferroni corrected.

Twenty tumors were evaluated by FDG PET (n = 10 in each treatment group). Uptake of FDG and FLT were determined relative to baseline at day 1, 3 and 10 after treatment initiation with everolimus (Figure 4).

By groups, no significant difference in SUV_mean_ or SUV_max_ FDG uptake was observed between the treatment and control group at any time. At day 10 FDG SUV_mean_ and SUV_max_ were significantly increased in the control group when compared to baseline (114±4%; P = 0.015 for SUV_mean_ and 113±3% P = 0.004 for SUV_max_) whereas no difference in FDG uptake was observed in the treatment group on day 10 when compared to baseline.

### Uptake of FLT in H727 Xenografts after Treatment with Everolimus

Sixteen tumors were evaluated by FLT PET (n = 8 in each treatment group).

On day 3 relative FLT SUV_mean_ and SUV_max_ uptake was significantly increased in both the control group (112±3%; P = 0.017 for SUV_mean_ and 112±3%; P = 0.024 for SUV_max_ and the everolimus group (116±4%; P = 0.012 for SUV_mean_ and 121±5%; P = 0.012 for SUV_max_) when compared to baseline. The relative FLT uptake in the control group was significantly raised on day 10 compared to baseline (116±4%; P = 0.015 for SUV_mean_ and 113±4%; P = 0.040 for SUV_max_) whereas no difference in FLT uptake was observed in the treatment group. At day 10, the relative FLT SUV_mean_ was significantly higher in the control group compared to the everolimus group (116±4% vs. 91±6%; P = 0.010) (figure 4).

### Correlations between PET and Tumor Growth

**Figure 5:**
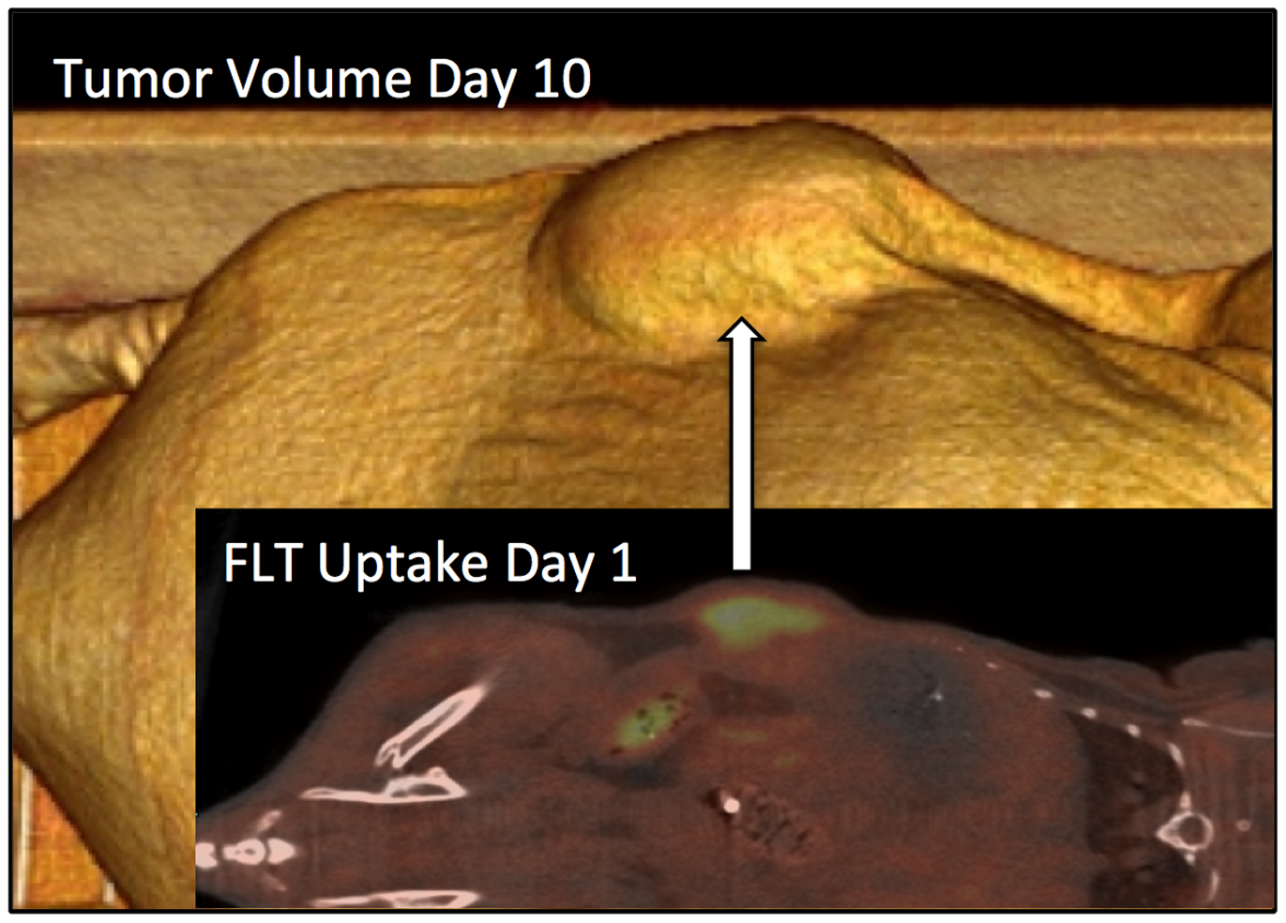
PET uptake and Tumorvolume. Comparison of early FLT PET on day 1 with late CT volume on day 10 in same animal. Correlations for the whole group between early PET and late CT volumes are shown in Figure 6.

**Figure 6:**
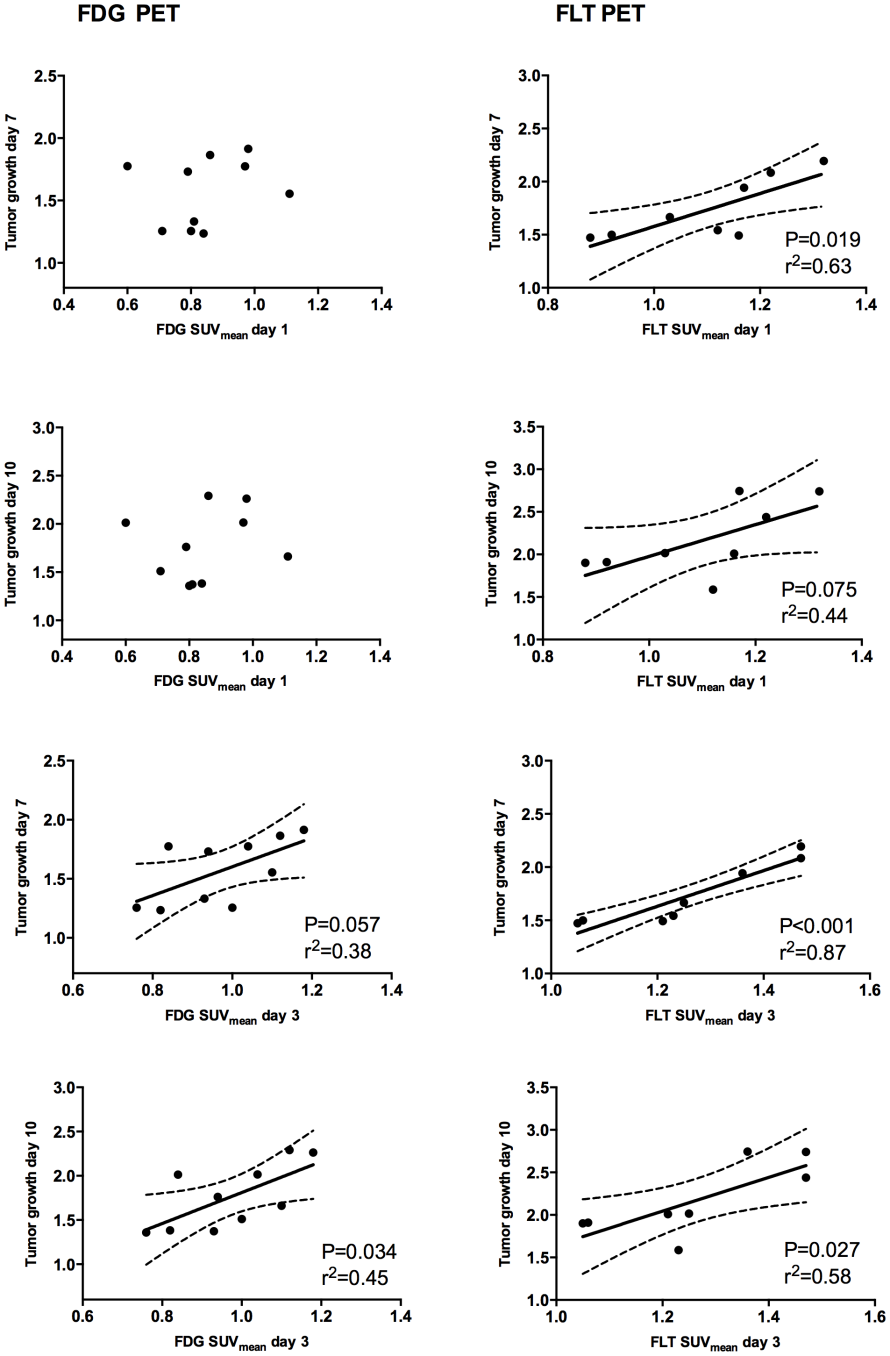
Early PET correlated to later tumorgrowth. Correlations of individual tumor uptake (SUVmean) day 1 and day 3 and subsequent tumor growth until day 7 and 10. Left panel: mean uptake of FDG at day 3 correlated significantly with tumor growth until day 10, (n = 10). Right panel: mean uptake of FLT at day 1 correlated significantly with tumor growth until day 7 and FLT uptake at day 3 correlated with tumor growth until both day 7 and 10 (n = 8 tumors). Tumor growth is expressed as volume relative to baseline, 95% confidence intervals are shown. Correlations with SUVmax show same trend (not shown).

The correlation between day 1 uptake of FLT PET and tumorvolume at day 10 is shown in Figure 5. All correlations as measured by early SUV_mean_ (day 1 and 3 ) and subsequent tumor growth (day 7 and 10) are shown for both FDG PET and FLT PET in Figure 6.

Tumor growth was measured as the relative increase in tumor size from baseline to either day 7 or day 10.

Within the everolimus treated group, FDG SUV_mean_ uptake day 1 did not correlate with tumor growth neither day 7 nor day 10. FDG SUV_mean_ uptake at day 3 correlated with tumor growth day 10 (r^2^ = 0.45; P = 0.034) and showed a trend towards correlation with day 7 (r^2^ = 0.38; P = 0.057).

SUV_mean_ of FLT within the everolimus treated group day 1 significantly correlated with tumor growth day 7 (r^2^ = 0.63; P = 0.019), and showed a trend toward correlation at day 10 (r^2^ = 0.44; P = 0.075). FLT SUV_mean_ uptake day 3 was significantly correlated with tumor growth day 7 (r^2^ = 0.87; P<0.001) and day 10 (r^2^ = 0.58; P = 0.027).

Correlations for SUV_max_ showed same trend (data not shown).

## Discussion

The major finding of our study was that everolimus inhibited tumor growth of the human lung carcinoid H727 and that FLT PET performed early after treatment initiation could predict later tumor growth.

In our study of human carcinoid lung NET xenografts, everolimus significantly inhibited tumor growth by almost 50% compared to placebo treated tumors, indicating an anti proliferative effect of everolimus on H727 NET.

In accordance with this, it has been found that mTOR activation is involved in the proliferation of subtypes of NETs in patients. In pancreatic NET cells, autocrine activation of the pathway is mediated through insulin-like growth factor 1 (IGF1) [34]. A gene expression profile study of sporadic pancreatic NETs revealed down regulation of TSC2 and PTEN, two key inhibitors of the AKT/mTOR pathway in most of the sporadic NETs. Furthermore, a correlation between low TSC2 and PTEN expression and an increased rate of cell proliferation and a shorter progression free and overall survival has been found [27].

A phase II trial among patients with progressive pancreatic NET disease showed promising results using everolimus or everolimus in combination with octreotide [35].

The subsequent phase III randomized double-blind placebo-controlled trial consisting of 410 patients with progressive well-differentiated pancreatic NET, found that the median progression free survival (PFS) was 11 months with everolimus and 4.6 months with placebo [36].

Based on these results, US and European health authorities approved the use of everolimus for advanced pancreatic NET.

Everolimus has also been evaluated in a large placebo-controlled phase III trial (RADIANT 2) in different types of NETs (small intestinal and lung) associated with the carcinoid syndrome (n = 429). PFS was prolonged by 5.1 months for patients treated with both everolimus and octreotide LAR (long acting somatostatin analogue) compared to patients receiving placebo and octreotide LAR. The trial documented that everolimus can be used in a broad spectrum of NET patients [37]. In a subgroup analysis of the RADIANT 2 trial, colorectal NETs were found to have a PFS of 29.9 months for the everolimus plus octreotde LAR treated group compared to 6.6 months on octreotide LAR alone [38]. Furthermore, a subgroup analysis of patients with advanced lung NET showed that the addition of everolimus to octreotide LAR improved the median PFS 2.4 fold [39]. This is consistent with our findings that everolimus inhibits the tumor growth in H727 human lung carcinoid xenografts.

*In vitro* studies have also shown that everolimus might be effective in the treatment of lung NETs. Accordingly, Zatelli et al. studied the effect of mTOR on bronchial carcinoids in 24 different bronchial carcinoids dispersed in cell cultures. In 15 of the cell cultures, everolimus significantly reduced cell viability, inhibited p706K activity and blocked IGF1 proliferative effects. The responding cell cultures were found to up-regulate mTOR expression indicating that bronchial carcinoids may be a suitable target for mTOR inhibition [40].

Although everolimus is effective in many NETs, the effect of this costly therapy is variable on an individual basis. Therefore, early detection of responders versus non-responders would be of great value. Since everolimus works by inhibiting the mTOR complex, an early detection of changes in mTOR pathway effects, like glycolysis and proliferation, might be visualized by FDG and FLT PET. To the best of our knowledge this has never been tested in NETs.

In our study of H727 NET we found that especially FLT was effective in early prediction of later tumor growth. Accordingly, FLT at day one, where no effect on tumor growth was present, could predict growth until day 7 (r^2^ = 0.63; P = 0.019). Clinically this would translate into that FLT PET early (days) after initiation of everolimus therapy in NET, could be used to identify responders and non-responders rather than waiting 6–8 weeks to see tumor shrinkage according to RECIST. In this way, non-responders could then early after treatment initiation be changed to alternative therapies and spared to undergo non-effective and expensive treatments. In support of this, non-NETs studies also indicate that FDG and FLT can be used to detect effect of everolimus: Accordingly, Nogová et al. used FDG for detecting the response of everolimus treatment in 8 non-small cell lung cancer patients. They scanned the patients at baseline and after 8 and 28 days of therapy and measured FDG by SUVmax and SUVmean [41]. In 5 patients there was a decrease in FDG uptake and the patient with the greatest reduction in FDG SUV had the longest PFS.

Others have described the use of FDG in a human gastric cell line to determine the optimal biological dose of everolimus soon after therapy initiation [30] and likewise, Aide et al. described the use of FLT as a surrogate marker of everolimus efficacy in a preclinical cisplatin resistant ovarian tumor model [42]. This is attractive since increasing mTOR dose beyond a certain level does not improve antitumor activity and may increase the incidence of adverse effects [43].

Wei et al. found that both FLT and FDG uptake decreased and reflected sensitivity towards mTOR inhibition in glioblastoma xenografts in *vivo* [44].

Ours findings that early imaging with FLT rather than FDG predicts later tumor volume is supported by other preclinical studies demonstrating that mTOR-inhibition is better predicted by FLT than FDG in lymphoma cell lines [45] and ovarian cancer cell lines [12]. Fuereder et al. also found FLT uptake to predict sensitivity to mTOR inhibition in gastric cancer cell lines but did not compare with FDG [46].

### Study Limitations

The present study is based on a single bronchial carcinoid cell line. Therefore, we cannot prove that the concept of early prediction of everolimus effect on tumor growth using FLT or FDG holds for all NETs. However, we believe the proof of concept data, are important and final testing will anyhow need to be performed in patients.

### Conclusion

We found that everolimus was effective in vivo in a human xenografts lung carcinoid NET and that FLT PET could effectively be used for early prediction of tumor growth. We suggest that FLT PET may be used for tailoring therapy in NETs with everolimus through early identification of responders and non-responders. In patients with FLT negative tumors FDG may represent an alternative. Whether such an algorithm translates clinically into better outcome remains to be tested.
